# Differential Effects of Treatment Strategies in Individuals With Chronic Ocular Surface Pain With a Neuropathic Component

**DOI:** 10.3389/fphar.2021.788524

**Published:** 2021-12-23

**Authors:** Sneh Patel, Rhiya Mittal, Elizabeth R. Felix, Konstantinos D. Sarantopoulos, Roy C. Levitt, Anat Galor

**Affiliations:** ^1^ Bascom Palmer Eye Institute, University of Miami Miller School of Medicine, Miami, FL, United States; ^2^ Surgical Services, Miami Veterans Affairs Medical Center, Miami, FL, United States; ^3^ Department of Physical Medicine and Rehabilitation, University of Miami Miller School of Medicine, Miami, FL, United States; ^4^ Department of Anesthesiology, Perioperative Medicine and Pain Management, University of Miami Miller School of Medicine, Miami, FL, United States; ^5^ John T. MacDonald Foundation Department of Human Genetics, University of Miami Miller School of Medicine, Miami, FL, United States; ^6^ John P. Hussman Institute for Human Genomics, University of Miami Miller School of Medicine, Miami, FL, United States

**Keywords:** ocular surface pain, cornea, dry eye disease, nociceptive pain, neuropathic pain, sensitization, central mechanisms, peripheral mechanisms

## Abstract

**Background:** Dysfunction at the ocular system via nociceptive or neuropathic mechanisms can lead to chronic ocular pain. While many studies have reported on responses to treatment for nociceptive pain, fewer have focused on neuropathic ocular pain. This retrospective study assessed clinical responses to pain treatment modalities in individuals with neuropathic component ocular surface pain.

**Methods:** 101 individuals seen at the University of Miami Oculofacial Pain Clinic from January 2015 to August 2021 with ≥3 months of clinically diagnosed neuropathic pain were included. Patients were subcategorized (postsurgical, post-traumatic, migraine-like, and laterality) and self-reported treatment outcomes were assessed (no change, mild, moderate, or marked improvement). One-way ANOVA (analysis of variance) was used to examine relationships between follow up time and number of treatments attempted with pain improvement, and multivariable logistic regression was used to assess which modalities led to pain improvement.

**Results:** The mean age was 55 years, and most patients were female (64.4%) and non-Hispanic (68.3%). Migraine-like pain (40.6%) was most common, followed by postsurgical (26.7%), post-traumatic (16.8%) and unilateral pain (15.8%). The most common oral therapies were α2δ ligands (48.5%), the m common topical therapies were autologous serum tears (20.8%) and topical corticosteroids (19.8%), and the most common adjuvant was periocular nerve block (24.8%). Oral therapies reduced pain in post-traumatic (81.2%), migraine-like (73%), and unilateral (72.7%) patients, but only in a minority of postsurgical (38.5%) patients. Similarly, topicals improved pain in post-traumatic (66.7%), migraine-like (78.6%), and unilateral (70%) compared to postsurgical (43.7%) patients. Non-oral/topical adjuvants reduced pain in postsurgical (54.5%), post-traumatic (71.4%), and migraine-like patients (73.3%) only. Multivariable analyses indicated migraine-like pain improved with concomitant oral α2δ ligands and adjuvant therapies, while postsurgical pain improved with topical anti-inflammatories. Those with no improvement in pain had a shorter mean follow-up (266.25 ± 262.56 days) than those with mild (396.65 ± 283.44), moderate (652 ± 413.92), or marked improvement (837.93 ± 709.35) (*p* < 0.005). Identical patterns were noted for number of attempted medications.

**Conclusion:** Patients with migraine-like pain frequently experienced pain improvement, while postsurgical patients had the lowest response rates. Patients with a longer follow-up and who tried more therapies experienced more significant relief, suggesting multiple trials were necessary for pain reduction.

## Introduction

The International Association for the Study of Pain (IASP) defines pain as a “an unpleasant sensory and emotional experience associated with, or resembling that associated with, actual or potential tissue damage.” (IASP Terminology, 2020) Ocular surface pain, one form of pain that is estimated to affect 5–30% individuals ≥50 years worldwide (Mehra et al., 2020), is often characterized by patients as “dryness”, “burning”, “aching”, or “tenderness”, among other terms. While ocular surface pain was initially lumped under the heading of “dry eye disease”, it is now recognized that pain can exist independently from tear dysfunction. Ocular surface pain can result from pathology at a number of sites including ongoing nociceptive issues at the level of the ocular surface and neuropathic mechanisms at the level of peripheral (e.g. cornea) or central nerves (Yu et al., 2011). In addition, nociceptive and/or neuropathic issues can occur in isolation or occur as part of a wider systemic disease (e.g. Sjögren’s, fibromyalgia, migraine) (Galor et al., 2016a; Diel et al., 2020). Beyond its prevalence, ocular surface pain is often chronic and is a major cause of disability and morbidity through its negative impact on quality-of-life via impaired social, physical, and mental functioning, leading to decreased productivity (Mertzanis et al., 2005; Patel et al., 2019).

Ocular surface pain is mediated via molecular and electrical signaling across activated neural pathways at various levels. Furthermore, while physiologic and neural processes are involved in the propagation of the pain signal, complex non-neural mechanisms, such as emotional and psychological factors, also play a role in the sensation of pain. Specifically, fast tear evaporation, corneal epithelial erosions, and ocular surface inflammation are common abnormalities that may contribute to chronic ocular surface pain. In addition, insults at the level of peripheral nociceptors (e.g. cornea and conjunctivae) or central nerves (e.g. trigeminal subnucleus caudalis, thalamus, or higher centers), can contribute to pain, including nerve injury associated with infection, trauma, chemical exposure, and metabolic disorders (Mehra et al., 2020). Finally, neuro-inflammatory, behavioral, cognitive and emotional mechanisms play a significant role in the perception and maintenance of pain and its manifestations, adding to the complexity of diagnosis and treatment of this common form of chronic pain.

As such, when approaching an individual with ocular surface pain, it is important to obtain a thorough history and complete ocular and neurologic examination for all potential contributors to this form of chronic pain. The examination typically begins with an evaluation of ocular surface abnormalities as potential sources for nociceptive pain. These include testing for tear film abnormalities (e.g. decreased tear production, high or unstable tear osmolarity, presence of inflammatory mediators), abnormal anatomy (e.g. conjunctivochalasis, pterygium), trauma and toxicity (e.g. topical glaucoma medications) as well as coexisting conditions (Mehra et al., 2020). Neuropathic pain is a clinical diagnosis and several findings suggest its presence, including symptoms out of proportion to signs of disease (Ong et al., 2018), a symptoms profile of sensitivity to wind and light (the ocular equivalents of hyperalgesia and allodynia) (Kalangara et al., 2017), abnormal corneal sensitivity (Galor et al., 2020), and persistent pain despite treatment of ocular surface abnormalities (Galor et al., 2016b). Furthermore, a centralized neuropathic component is suggested if pain persists despite placement of topical anesthesia on the ocular surface (Crane et al., 2017a), or when individuals report pain to light touch around the eye (consistent with presence of tactile allodynia or secondary hyperalgesia) (Timmerman et al., 2014). Overall, this complexity highlights the need for patient-centered, comprehensive, multidisciplinary approach and multimodal therapies to best address chronic ocular surface pain. This area of study represents untapped potential in ophthalmology and pain medicine, as creating new ways of precisely diagnosing and categorizing a patient’s pain could lead to novel pathways for guiding therapeutic decision-making.

Generally, nociceptive pain is targeted through use of topical therapies, while neuropathic pain can be treated with oral agents or adjunctive therapies if treatment of nociceptive pain fails and/or a neuropathic component is highly suspected. While many studies have examined treatment outcomes for nociceptive sources of ocular pain (Dermer et al., 2020; Mittal et al., 2021), fewer have examined outcomes after treatment of neuropathic ocular pain. Furthermore, available literature typically report on the effects of one therapeutic modality in a limited number of patients (Ozmen et al., 2020). To improve our fund of knowledge, this study examined clinical data from a cohort of individuals with a presumed neuropathic component to their chronic ocular surface pain, with the aim of studying subjective clinical responses to a number of commonly utilized medications.

## Materials and Methods

### Study Population

We identified 124 individuals who sought care at the University of Miami Oculofacial Pain Clinic (Bascom Palmer Eye Institute and/or the University of Miami Pain Management Clinic) between January 2015 and August 2021 and whose medical records contained a diagnosis of ocular pain (International Classification of Diseases 10 [ICD10], code H57.XX). Patients were included if they had unilateral or bilateral pain for a duration ≥3 months, with a presumed neuropathic component. The diagnosis of neuropathic ocular pain was made clinically by the treating physician based on the presence of one or more pain features that included: sensitivity to wind and light (Crane et al., 2017b; Kalangara et al., 2017), symptoms out of proportion to ocular surface signs (Ong et al., 2018), abnormal corneal sensitivity (Spierer et al., 2016), persistent pain after topical anesthetic (Crane et al., 2017a), and cutaneous allodynia around the eye. Exclusion criteria included individuals whose pain lasted <3 months, or whose pain resolved with treatment of nociceptive sources of pain (e.g. topical anti-inflammatory agents, surgical correction of anatomic abnormality, etc.). After consideration of these criteria, 101 individuals remained in the study for analysis. This retrospective review was approved by the University of Miami Institutional Review Board and followed the tenets of the Declaration of Helsinki.

### Data Collection

For each subject, electronic medical record information was collected including demographics (age, gender, race, ethnicity) and clinical (past ocular, medical, and surgical history) variables. Additionally, co-morbid conditions particularly those related to chronic systemic pain (e.g. fibromyalgia, peripheral neuropathy, trigeminal neuralgia, migraine) were recorded, as was information regarding prior or current ocular pain treatments, including the use of oral neuromodulators (e.g., α2δ ligands, tricyclic and serotonin-norepinephrine reuptake inhibitors [SNRI]), topical ocular therapies (e.g., anti-inflammatory therapies, autologous serum tears), and non-oral/topical adjuvant treatments (e.g., trigeminal nerve stimulation [TNS] and interventional procedures (botulinum toxin injection, steroid-anesthetic based periocular nerve block, or sphenopalatine or superior cervical ganglion block). Time to follow up from first to last visit was also calculated in days.

### Ocular Pain Characteristics and Pain Groups

Data on pain characteristics was collected including temporality, location (unilateral vs bilateral), descriptors (e.g., squeezing, burning, throbbing, pressure, foreign body sensation), and triggers (sensitivity to light or photophobia, cutaneous allodynia). Based on pain history and characteristics, patients were placed into one of four subcategories. The Postsurgical Pain group included those who developed ocular pain after undergoing surgery (e.g. refractive, cataract, other procedure). The Post-Traumatic Pain group included individuals whose pain began after a non-surgical trauma (chemotherapy, radiation, traumatic brain injury). The Migraine-like Pain group included individuals with bilateral pain that started spontaneously and was accompanied by photophobia, with many of these individuals having co-morbid migraine or headache syndromes. The Unilateral Pain group included individuals with spontaneous unilateral pain that did not start after surgery and was not typical for trigeminal neuralgia but none-the-less had neuropathic qualities, as outlined above.

### Treatment Outcomes

Treatment outcomes were determined by examining patient subjective responses after starting a given pain modulating therapy (e.g., comparison to an established baseline pain level), graded on a scale of “no change” (no change), “mild improvement” (some alleviation of symptoms), “moderate improvement” (great improvement but persistence of minor symptoms), or “marked improvement” (resolution or near-resolution of pain).

### Statistical Analyses

Analyses were performed using SPSS 22.0 (IBM SPSS Statistics for Windows, 2013). Descriptive statistics were used to summarize demographic and clinical information within the population and each pain subcategory. Information on response to treatment (improvement in pain with treatment) was collected in a binary (yes or no) and scaled (none, mild, moderate, or marked improvement) fashion, and compared between ocular pain subgroups as outlined above. One-way ANOVA (analysis of variance) was utilized to examine differences in mean clinical follow-up time as well as number of attempted oral, topical, and adjuvant medications across pain improvement groups (none, mild, moderate, or marked). Finally, individual multivariable logistic regressions models were created for each pain subgroup using the binary variable ‘*Clinical Improvement in Pain’* as the outcome to assess which modalities were clinically effective when utilized concomitantly.

## Results

### Study population and Demographics

The study population consisted of 101 individuals who met inclusion and exclusion criteria. The mean age was 55 years, and most patients were female (64.4%), white (92.1%), and non-Hispanic (68.3%). Several systemic comorbidities were noted, including chronic joint pain (27.7%), migraine (24.8%), and fibromyalgia (7.9%). All individuals fit into one of the ocular pain subcategories, with migraine-like pain (40.6%) being most common, followed by postsurgical pain (26.7%), which most often occurred after refractive surgery, and finally post-traumatic pain (16.8%) and unilateral pain (15.8%). The most common pain descriptor was throbbing/shooting pain (19.8%), and many individuals reported photophobia (49.5%) as a pain trigger, as well as pain to light touch around the eye (cutaneous allodynia, 19.8%) (Table 1).

**TABLE 1:** Demographics, medical comorbidities, and pain characteristics, by population and by pain subgroup.

	All patients; n (%)	Postsurgical pain; n (%)	Post-traumatic pain; n (%)	Migraine-like pain; n (%)	Unilateral pain; n (%)
	101 (100%)	27 (26.7%)	17 (16.8%)	41 (40.6%)	16 (15.8%)
Demographics					
Age (mean, SD; years)	55 (17)	54 (18)	53 (18)	52 (17)	61 (15)
Gender, female	65 (64.4%)	17 (63.0%)	14 (82.4%)	25 (61.0%)	9 (56.3%)
Race, White	93 (92.1%)	24 (88.9%)	16 (88.9%)	39 (95.1%)	14 (87.5%)
Ethnicity, Hispanic	31 (30.7%)	8 (29.6%)	5 (29.1%)	11 (26.8%)	7 (43.8%)
Medical Comorbidities					
Chronic joint pain	28 (27.7%)	1 (3.7%)	7 (41.2%)	15 (36.6%)	5 (31.3%)
Fibromyalgia	8 (7.9%)	2 (7.4%)	0 (0)	4 (9.8%)	2 (12.5%)
Migraine	25 (24.8%)	6 (22.2%)	2 (11.8%)	16 (39.0%)	1 (6.3%)
Peripheral neuropathy	4 (4.0%)	3 (11.1%)	0 (0)	0 (0)	1 (6.3%)
Trigeminal neuralgia	7 (6.9%)	2 (7.4%)	0 (0)	2 (4.9%)	3 (18.8%)
Herpetic neuralgia	5 (5.0%)	0 (0)	2 (11.8%)	2 (4.9%)	1 (6.3%)
Ocular History					
Pain >1 year	93 (92.1%)	24 (88.9%)	17 (100%)	38 (92.7%)	14 (87.5%)
Post-LASIK	9 (8.9%)	9 (33.3%)	0 (0)	0 (0)	0 (0)
Post-PRK	2 (2.0%)	2 (7.4%)	0 (0)	0 (0)	0 (0)
Post-CE/iol	5 (4.9%)	5 (19.0%)	0 (0)	0 (0)	0 (0)
Pain Triggers and Descriptors					
Photophobia	50 (49.5%)	11 (40.7%)	3 (17.6%)	36 (87.8%)	0 (0)
Cutaneous Allodynia^a^	20 (19.8%)	4 (14.8%)	7 (41.2%)	7 (17.1%)	2 (12.5%)
Paresthesia (tingling)	9 (8.9%)	2 (7.4%)	0 (0)	6 (14.6%)	1 (6.3%)
Foreign Body Sensation	10 (9.9%)	3 (11.1%)	4 (23.5%)	2 (4.9%)	1 (6.3%)
Dull pain	4 (4.0%)	0 (0)	2 (11.8%)	1 (2.4%)	1 (6.3%)
Throbbing/Shooting pain	20 (19.8%)	7 (25.9%)	1 (5.8%)	9 (22%)	3 (18.8%)

a Pain on light touch of the skin around the eye.

SD = standard deviation; LASIK = laser-assisted *in situ* keratomileusis; PRK = photorefractive keratectomy; CE/iol = cataract extraction and intraocular lens.

### Subjective Response to Various Treatment Modalities Across Pain Subgroups

A variety of modalities were attempted (Table 2). The most common oral medications were α2δ ligands (48.5%), non-steroidal anti-inflammatory drugs (NSAIDs, 31.7%), and serotonin-norepinephrine reuptake inhibitors (SNRIs, 16.8%). Oral medications were commonly paired with topical therapy, such as autologous serum tears (AST, 20.8%) and/or a topical anti-inflammatory (e.g. topical steroid [19.8%], cyclosporine or lifitegrast [17.8%], or less commonly tacrolimus [8.9%]). Finally, a minority of patients received adjuvant therapies, like trigeminal nerve stimulation (TNS, 15.8%), steroid-anesthetic based periocular nerve block (24.8%), and/or botulinum toxin injections (10.9%).

**TABLE 2:** Utilized Oral, Topical, and Adjuvant Therapies, by Population and by.

	All patients (n, % of population	Postsurgical (n, % of subgroup)	Post-traumatic (n, % of subgroup)	Migraine-like (n, % of subgroup)	Unilateral (n, % of subgroup)
Oral Agents	90 (89.1%)	26 (96.3%)	16 (94.1%)	37 (90.2%)	11 (68.8%)
Pregabalin/Gabapentin	49 (48.5%)	16 (59.3%)	11 (64.7%)	17 (41.5%)	6 (37.5%)
TCA (amitriptyline)	9 (8.9%)	3 (11.1%)	3 (17.6%)	0 (0)	3 (18.8%)
SNRI (duloxetine)	17 (16.8%)	5 (18.5%)	3 (17.6%)	6 (14.6%)	2 (12.5%)
Anticonvulsant (topiramate)	9 (8.9%)	3 (11.1%)	1 (5.9%)	3 (7.3%)	2 (12.5%)
Acetaminophen	17 (1.8%)	5 (18.5%)	3 (17.6%)	7 (17.1%)	2 (12.5%)
Any NSAID^a^	32 (31.7%)	6 (22.2%)	7 (41.2%)	12 (29.3%)	7 (43.8%)
Any muscle relaxant^b^	32 (31.7%)	1 (3.7%)	2 (11.8%)	26 (63.4%)	2 (12.5%)
Any opioid agonist/antagonist^c^	15 (14.9%)	3 (11.1%)	3 (17.6%)	5 (12.2%)	4 (25%)
Topical Agents	52 (51.5%)	16 (59.3%)	12 (70.6%)	14 (34.2%)	10 (62.5%)
AST	21 (20.8%)	8 (29.6%)	5 (29.4%)	3 (17.1%)	5 (31.3%)
Topical corticosteroid	20 (19.8%)	3 (11.1%)	3 (17.6%)	6 (14.6%)	8 (50%)
Topical cyclosporine, lifitegrast	18 (17.8%)	7 (25.9%)	2 (17.6%)	6 (14.6%)	3 (18.8%)
Topical tacrolimus	9 (8.9%)	1 (3.7%)	3 (17.6%)	3 (7.3%)	2 (12.5%)
Adjuvant Agents	39 (38.6%)	11 (40.7%)	7 (41.2%)	15 (36.6%)	6 (37.5%)
TNS	16 (15.8%)	5 (18.5%)	1 (5.9%)	9 (22%)	1 (6.3%)
Peri-ocular nerve block	25 (24.8%)	9 (33.3%)	5 (29.4%)	6 (14.6%)	5 (31.3%)
Ganglion block	6 (5.9%)	1 (3.7%)	1 (5.9%)	1 (2.4%)	3 (18.8%)
Botulinum injection	11 (10.9%)	0 (0)	0 (0)	10 (24.4%)	1 (6.3%)

a Ibuprofen, Diclofenac, Meloxicam, celecoxib.

b Baclofen, Cyclobenzaprine.

c Tramadol, Naltrexone, Oxycodone.

TCA = tricyclic antidepressant; SNRI = serotonin-norepinephrine reuptake inhibitor; NSAID = Non-Steroidal Anti-Inflammatory Drug; AST = autologous serum tears; TNS = trigeminal nerve stimulation.

**FIGURE 1:**
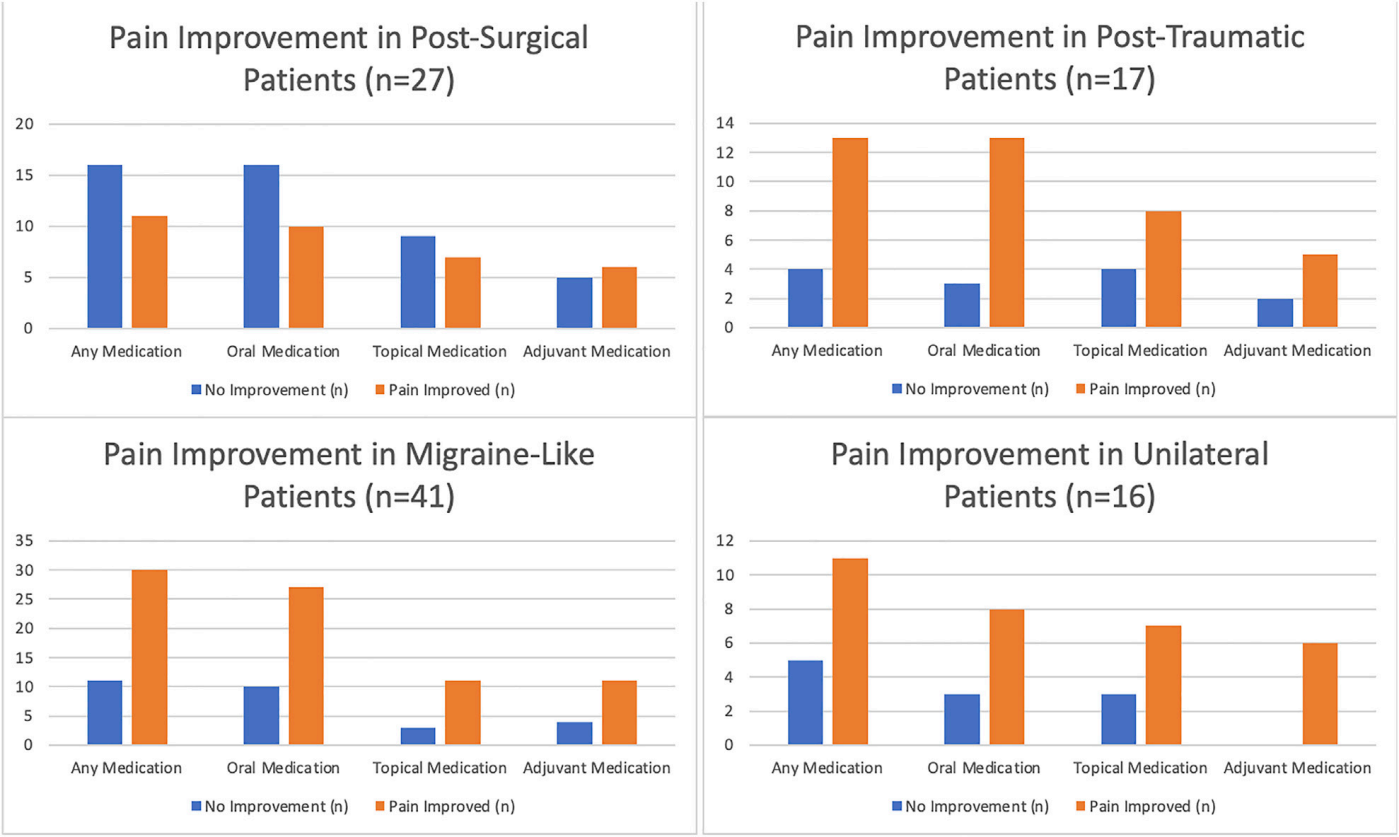
Pain response to various treatment strategies in patients with neuropathic pain, by underlying cause.

Figure 1 and Table 3 (and Supplementary Tables 1–4, Appendix**)** outline response to therapy, by pain subgroups. At least one oral medication reduced pain to a mild or greater degree in the majority of post-traumatic (81.2%), migraine-like (73%), and unilateral pain (72.7%) groups but in the minority of postsurgical pain patients (38.5%). Marked improvement with oral medications was most frequently noted in migraine-like patients (21.6%) compared to the other groups (postsurgical 15.4%, post-traumatic 12.5%, unilateral 0%). In a similar manner, topical medication more frequently led to a subjective improvement in pain in the post-traumatic (66.7%), migraine-like (78.6%), and unilateral (70%) groups compared to the postsurgical group (43.7%). Again, marked improvement was most common in the migraine-like group (21.4%) followed by the postsurgical group (18.8%), then the post-traumatic (8.3%) and unilateral (0%) groups. Finally, the use of one or more adjuvants reduced pain to a mild or greater degree in 54.5% of the postsurgical, 71.4% of the post-traumatic, 73.3% of the migraine-like, and 0% of the unilateral groups. Marked improvement in pain after adjuvant use was most common in the migraine-like group (20%) followed by the postsurgical group (11.1%), while in the other two groups these therapies did not lead to marked improved of pain (0%, each).

**TABLE 3:** Proportion of medications that led to improvement in pain, by pain subgroup.

	Pain improvement in response to treatment	
	None (n; % of taking)	Any (n; % of taking)
Postsurgical (*n* = 27)		
Any medication	16 (59.3%)	11 (40.7%)
Oral medications	16 (61.5%)	10 (38.5%)
Topical medications	9 (56.3%)	7 (43.8%)
Adjuvant therapies	5 (45.5%)	6 (54.5%)
Post-traumatic (*n* = 17)		
Any medication	4 (23.5%)	13 (76.5%)
Oral medications	3 (18.8%)	13 (81.3%)
Topical medications	4 (33.3%)	8 (66.7%)
Adjuvant therapies	2 (28.6%)	5 (71.4%)
Migraine-like (*n* = 41)		
Any medication	11 (26.8%)	30 (73.2%)
Oral medications	10 (27%)	27 (73%)
Topical medications	3 (21.4%)	11 (78.6%)
Adjuvant therapies	4 (26.7%)	11 (73.3%)
Unilateral (*n* = 16)		
Any medication	5 (31.3%)	11 (68.7%)
Oral medications	3 (27.3%)	8 (72.7%)
Topical medications	3 (30%)	7 (70%)
Adjuvant therapies	0 (0%)	6 (100%)

*n* = number in the group.

### Relationship Between Subjective Pain Improvement and Follow-Up Time

Next, the relationship between follow-up time (days between initial and most recent visit) and number of medications attempted across patients with differing subjective responses to treatment were examined. Individuals who experienced no improvement had a shorter follow up time (mean = 266.25 days, SD = 262.56, range = 897) compared to those with mild (mean = 396.65, SD = 283.44, range = 1227), moderate (mean = 652, SD = 413.92, range = 1342), or marked (mean = 837.93, SD = 709.35, range = 2,222) improvement in pain. Via ANOVA, there were significant differences in mean follow-up between those with improvement and those without (*p* < 0.005). Subgroup testing also indicated that follow-up time for those with none or mild improvement in pain were non-significantly different, while those with moderate or marked improvement in pain had significantly longer follow-up periods with a clinician. Analyses further showed that patients who experienced improvement in pain tried more medications, suggesting that multiple trials were necessary to achieve increasing pain control (Table 4).

**TABLE 4:** *Population-wide Differences in Mean Follow-up Time and Medications Attempted Between Different Categories of Clinical Improvement with Treatment*.

	None	Mild	Moderate	Marked	*p*-value
FU time (days), mean ± SD	266.25 ± 262.56	396.65 ± 283.44	652 ± 413.92	837.93 ± 709.35	<0.005
Number oral meds tried, mean ± SD	1.36 ± 1.05	1.62 ± 0.85	1.67 ± 1.24	1.70 ± 0.90	0.02
Number of topical meds tried, mean ± SD	1.09 ± 1.12	1.13 ± 1.03	1.33 ± 1.05	1.30 ± 1.14	<0.005
Number of adjuvant meds tried, mean ± SD	0.44 ± 0.74	0.68 ± 0.84	0.73 ± 0.88	0.8 ± 0.67	<0.005
Number of any meds tried, mean ± SD	3.05 ± 1.50	3.32 ± 1.22	3.67 ± 1.63	3.71 ± 1.42	0.05

FU = follow-up; SD = standard deviation.

### Multivariable Analysis of Effects of Multiple Treatments on Subjective Pain Improvement and Pain Triggers

Utilizing stepwise multivariable logistic regression analyses, we examined relationships between various treatments (independent variables) to ***any*** improvement in pain (dependent variable) in our pain subgroups. In postsurgical patients, topical cyclosporine/lifitegrast remained associated with improvement in pain (odds ratio (OR) = 1.31, 95% confidence interval (95%CI) 1.03–1.33, *p* = 0.04). Several treatments were predictive of pain improvement in the migraine-like group, including oral α2δ ligands (OR = 2.74, 95%CI 2.73–2.96, *p* = 0.02), muscle relaxants (OR = 1.36, 95%CI 1.33–1.37, *p* < 0.005), and TNS (OR = 1.20, 95%CI 1.19–1.21, *p* < 0.005). Examining these relationships with respect to pain triggers, in individuals with photophobia, oral α2δ ligands (OR = 2.18, 95%CI 1.78–2.21, *p* = 0.05) and muscle relaxants (OR = 1.32, 95%CI 1.31–1.34, *p* < 0.005) remained in the model, while in individuals with cutaneous allodynia, oral α2δ ligands (OR = 1.79, 95%CI 1.76–1.80, *p* < 0.005) and topical cyclosporine/lifitegrast (OR = 1.13, 95%CI 1.11–1.18, *p* < 0.005) remained in the model.

## Discussion

To summarize, we examined subjective responses to various therapies in individuals with chronic ocular surface pain with a neuropathic component. We found that despite the heterogeneity of patients, all fit into one of four pain subgroups, and that responses to treatment varied across groups, although there was significant variability within the groups. Overall, individuals with migraine-like pain reported the most frequent pain improvement (73.2%), generally with a combination of oral (α2δ ligands) and adjuvant (TNS) therapies, while the postsurgical group had the lowest overall response rate (40.7%) to the various therapies. This highlights the need for further studies to investigate other, more appropriate therapies to target the pain in the latter population. Furthermore, we found that the likelihood and degree of pain improvement increased with longer follow up time and with the number of medications utilized, indicative of inter-individual variability that necessitated multiple trials of medications to find a combination that led to clinical improvement. Given this current reality, it is essential to appropriately counsel patients on the trial-and-error approach and time frame needed to achieve clinical improvement in order to avoid early termination of care (Goyal and Hamrah, 2016).

We used various therapies in multiple compartments (oral, topical, adjuvant) due to the multiple potential locations of nerve dysfunction in our patient population (Mehra et al., 2020). Beyond nociceptive causes, peripheral (corneal) nerve abnormalities may contribute to pain in some individuals (Galor et al., 2018a). Confocal microscopy is one tool that can detect corneal nerve abnormalities (e.g. density, length, tortuosity) in individuals with chronic ocular surface pain (Patel et al., 2020; Patel et al., 2021). In one study of 16 individuals with presumed corneal neuropathic pain (9 of 16 due to postsurgical pain after refractive surgery]), low nerve count (10.5 ± 1.4 vs 28.6 ± 2.0 nerves/frame; *p* < 0.0001) and length (10,935.5 ± 1264.3 vs 24,714.4 ± 1056.2 μm/mm^2^; *p* < 0.0001) were noted compared to 12 healthy controls. Treatment with AST (20%; mean duration 3.8 ± 0.5 months, range 1–8 months) decreased pain in all individuals (mean 3.1 ± 0.3 vs baseline 9.1 ± 0.2; 0–10 scale; *p* < 0.0001) and increased nerve count (to 15.1 ± 1.6; *p* < 0.0001) and length (to 17,351.3 ± 1395.6 μm/mm^2^; *p* < 0.0001) (Aggarwal et al., 2019). Overall, in our study, 62.5% (5 of 8) of postsurgical patients had mild or greater improvement with serum tears, with three of 5 (60%) reporting marked improvement.

In addition to corneal nerve abnormalities, peripheral (trigeminal non-corneal) afferents may contribute to chronic ocular surface pain (Galor et al., 2018a). Several strategies can be used to address these potential abnormalities, including TNS, nerve blocks, and botulinum toxin (Mehra et al., 2020). TNS is a non-pharmacological approach that is often used in patients with migraine; the device generates impulses at the supratrochlear and supraorbital branches of trigeminal V1 via an adhesive electrode on the head (Zayan et al., 2020; Mehra et al., 2021). Supporting the use of TNS in patients with comorbid migraine and ocular pain, an American study of 18 individuals with severe ocular pain who utilized TNS for 6 months (3.7 ± 1.9 sessions/week at month 1, 2.7 ± 2.3 sessions/week at month 6) noted lower ocular pain intensity scores at 6 months compared to baseline (3.8 ± 3.5 to 2.7 ± 3.0, *p* = 0.02, a 31.4% reduction in pain). On subgroup analyses, individuals with comorbid migraine (n = 10) had a better response than those without co-morbid migraine, but all individuals experienced pain improvement to at least a moderate level (∼31.4%). Interestingly, pain improvement with TNS took time, with significant differences first noted 3 months after initiation of therapy (Mehra et al., 2021). A similar pattern emerged in a randomized placebo controlled study of TNS in migraine, highlighting that nerve modulatory therapies take time to translate into improvements in clinical manifestations (Chou et al., 2018). These findings are similar to our current study, where 66.7% (6 of 9) of individuals with migraine-like pain experienced pain improvement with TNS (33.3% mild, 33.3% moderate or greater).

Combination nerve blocks, consisting of a local anesthetic acting as a sodium channel inhibitor (for prevention of ectopic action potential generation) and long-acting corticosteroid (for potentiation of effect and additional mechanisms), have been commonly used to treat pain in an isolated anatomical area due to neuralgia (pain arising from a nerve) (Scholz et al., 1998; Galor et al., 2018a). In a case series of 11 subjects with chronic ocular pain with a presumed neuropathic component (3 migraine-like, seven postsurgical, two post-traumatic, 1 unilateral), seven experienced pain relief after nerve blockade (4 ml of 0.5% bupivacaine with 1 ml of 80 mg/ml methylprednisolone acetate), varying from hours to 7 months. This intervention was most effective in individuals with postsurgical (5 of 6) and unilateral pain (1 of 1) compared to the other pain types (0 of two migraine-like, 0 of one post-traumatic) (Small et al., 2020). Our current results reinforce these findings but in our study, all pain group types had a reasonable frequency of response to therapy, with any improvement noted most frequently in the post-traumatic (5 of 5), migraine-like (5 of 6), and unilateral (5 of 5) groups, followed by the postsurgical (4 of 9) group. Per our results, individuals in the postsurgical and migraine-like pain groups most frequently experienced moderate or greater relief (3 moderate or greater, each).

Botulinum toxin injection is another adjuvant therapy often applied to chronic ocular pain, being most frequently utilized in patients with migraine, with studies generally reporting a mild to moderate improvement in ocular symptoms after treatment (Johnson, 2007; Diel et al., 2018; Venkateswaran et al., 2020a). For example, an American study of 76 patients with chronic migraine who received BoNT-A toxin injections (100–150 U) reported a significant decrease in interictal photophobia scores (3.37 ± 2.54 from 4.89 ± 2.97, *p* < 0.001, range 0–10) after treatment (mean FU of 30.5 ± 7.65 days, range 19–56 days) (Diel et al., 2019). A similar reduction in interictal photophobia (5.27 ± 2.73 from 7.91 ± 2.05, *p* < 0.001, range 0–10) was noted in another American study of 117 patients with chronic migraine who received BoNT-A toxin injection (Diel et al., 2018). The migraine BoNT-A has been modified and used in individuals with neuropathic ocular pain but *without* a history of migraine. Four individuals treated with one session of BoNT-A (35 U given across seven forehead sites) reported a decrease in photophobia severity (3.25 ± 0.4 from 4.8 ± 0.4, range 0–5) and ocular discomfort (2.25 ± 1.0 from 4.5 ± 0.6, range 0–5) at 1 month follow-up (Venkateswaran et al., 2020b). In our current study, eight of 10 migraine-like pain patients who received botulinum toxin injections reported a subjective improvement in ocular pain (4 mild, four moderate). In addition, one patient in the unilateral pain group also experienced mild improvement in pain with the modified BoNT-A protocol.

A centralized component to pain may be suspected when chronic ocular surface pain is accompanied by photophobia, by cutaneous allodynia, and/or persistent pain after anesthesia applied onto ocular surface (Digre and Brennan, 2012). For individuals with centralized nerve pain, oral medications are a first line treatment. Commonly used oral neuromodulating agents include α2δ ligands (gabapentin or pregabalin), SNRIs (duloxetine), and TCAs (nortriptyline) (Patel et al., 2020). Such agents have a slow onset of action, with clinical effects often becoming apparent weeks to months after initiation (Mehra et al., 2020), something that further highlights the need for longer follow-up times and persistent therapies. Several case series have examined the effects of oral medications on chronic ocular surface pain–for example, in a case series of eight individuals (n = 4 postsurgical), gabapentin (starting 300 mg daily, escalation to 600–900 TID) and pregabalin (starting 75 mg daily, escalation to 150 mg BID) led to complete relief in two subjects (NRS = 0 on a 0–10 scale), marked relief in three subjects (NRS ≤2), and mild relief in one subject (NRS = 10 to 7), while two had no improvement in pain. Interestingly, the two subjects who noted complete relief were on concomitant SNRI (duloxetine; starting 20 mg, escalation to 60 mg daily) (Small et al., 2020). These findings are similar to our analyses, which indicated that a similar proportion of individuals in the postsurgical pain group had mild or greater improvement to an α2δ ligand (n = 6 of 10; 60%), four of which had a marked improvement in pain.

A similar effect has been noted with TCAs. A British study examined 25 individuals with peripheral neuropathic pain (neuropathic symptoms and IVCM findings e.g. presence of microneuromas) who were treated with nortriptyline (10–25 mg starting dose, escalation to 100 mg daily). Pain levels 4 weeks post-treatment were ∼60% lower than pre-treatment (NRS; 3.80 ± 2.39 vs 6.36 ± 2.18, *p* < 0.0001). Overall, 84% of subjects (*n* = 21) reported pain improvement [28% with >50% improvement (*n* = 7), 40% with 25–50% improvement (*n* = 10), and 32% with <25% improvement (*n* = 8)] (Ozmen et al., 2019). Because this study did not break down its population by etiology, and due to the low proportion of individuals utilizing TCAs in our population, comparisons to this study are difficult. Nonetheless, in our study, improvement in pain was rated as mild or moderate in five of nine individuals who attempted a TCA (n = 3 post-traumatic and n = 2 unilateral).

Low dose oral opioid antagonists (low dose naltrexone) have also been studied in centralized pain, with effects attributed to antihyperalgesia (Jackson et al., 2021) (transient blockade of µ- and δ opioid receptors) as well as reduced neuroinflammation (antagonistic binding to the Toll-like receptor-4) (Bostick et al., 2019). An American study of 59 patients (*n* = 14 postsurgical) with centralized neuropathic ocular pain (defined by presence of neuropathic symptoms, IVCM findings, and/or persistent pain after topical anesthetic) examined the effects of naltrexone 4.5 mg nightly (mean 14.87 ± 11.25 months) on chronic ocular surface pain. Overall, a 49.2% improvement in pain was noted from baseline (3.23 ± 2.60 from 6.13 ± 1.93, *p* < 0.001, range 0–10) (Dieckmann et al., 2021). While we grouped individuals utilizing any opioid agent into one category, naltrexone was the most common agent used; 15 individuals attempted any opioid medication in our population, and improvement in pain was seen in 10 of these patients (*n* = 3 post-traumatic, *n* = 3 migraine-like, n = 4 unilateral).

Finally, while less frequently studied, dysfunction at the autonomic nervous system may contribute to chronic ocular surface pain (Galor et al., 2018b). Along with the trigeminal nerve’s sensory input, the sympathetic nervous system (SNS) projects fibers to the cornea from the superior cervical ganglion, while the parasympathetic nervous system (PSNS) sends fibers from the ciliary ganglion (Galor et al., 2018b). Autonomic dysfunction contributes to a variable degree to chronic pain conditions, like fibromyalgia (Janzen and Scudds, 1997), cluster headaches (Costa et al., 2000; Pipolo et al., 2010), and complex regional pain syndrome (Quevedo et al., 2005). In patients with parasympathetic or sympathetic contributors to pain, sphenopalatine ganglion or superior cervical ganglion blocks respectively, and/or nerve stimulation as well as intrathecal delivery of analgesic agents have been used with some success. In particular, one case report of a patient with intractable post-refractive surgery (LASIK) pain was treated initially with a trigeminal nerve stimulator and later on with intrathecal bupivacaine-fentanyl delivery. The patient has reported stable pain since 2014 with >50% (moderate) pain relief for over a year (Hayek et al., 2016). In our study, six individuals (*n* = 3 unilateral vs. *n* = 1 postsurgical, post-traumatic, migraine-like each) received a block at the aforementioned ganglia, and all patients experienced improved pain except one postsurgical patient; among those who improved, the blocks most commonly led to mild (*n* = 3) and marked (*n* = 2) improvement in pain.

As with all studies, our findings must be considered bearing in mind the study limitations, which included a retrospective evaluation of multiple therapies in a wide range of individuals with chronic ocular surface pain from varied etiologies. Yet, this weakness is also a strength considering its originality, as prior studies have only examined the effect of one therapy in a particular patient population. In reality, the majority of patients with chronic ocular surface pain will receive a number of oral, topical, and adjuvant therapies that often work concomitantly. Another limitation is sample size considerations, especially when examining pain subgroups (e.g. unilateral). As such, future studies with larger populations are needed to validate the findings of our study. Furthermore, unaccounted confounders may have affected our data, such as emotional and psychosocial contributors to pain (Lamb et al., 2010; Otis et al., 2013; Patel et al., 2019). Other studies have demonstrated that targeting these aspects with a variety of therapies, such as cognitive behavior therapy, acupuncture, and exercise, can reduce pain intensity beyond medical therapy alone (Mehra et al., 2020) and as such, these factors should be examined in future studies. This is particularly pertinent to our findings, since cognitive modification and positive counseling may enhance compliance and motivate patients to remain compliant and persistent in maintaining their continuity of care and follow ups for as long as necessary to find an efficacious treatment approach. Finally, comparison to other studies is limited considering the varying populations and pain assessments utilized.

## Conclusion

Despite the study’s limitations, our study presents clinical outcomes in a wide range of patients with chronic ocular surface pain, treated with a variety of oral, topical, and adjuvant therapies. Overall, there was individual variability in treatment response, although some trends were noted by pain subgroup. One likely contributor to variability is our inability to pinpoint the location(s) of nervous system dysfunction (peripheral corneal, peripheral non-ocular, central, autonomic) for each patient. Even in patients with suspected central pain, the optimal combination of oral, topical, and adjuvant therapies is not known. In our population, some patient who failed treatment with an α2δ ligand, subsequently reported subjective pain reduction with a TCA or topiramate. This points to the necessity of a trial-and-error approach, which is currently widely utilized when treating individuals with chronic ocular surface pain. Our findings point to needed areas of future research, including the development of diagnostic tests that can localize nervous system abnormalities, and then application of personalized approaches that target these abnormalities with medications or other therapies that provide faster acting pain relief than currently available neuromodulators.

## Data Availability

The raw data supporting the conclusion of this article will be made available by the authors, without undue reservation.
