# Supplementary material for: Differential Effects of Treatment Strategies in Individuals With Chronic Ocular Surface Pain With a Neuropathic Component
Source: Front Pharmacol. 2021 Dec 23;12:788524. doi: 10.3389/fphar.2021.788524 (PMC8733738; doi:10.3389/fphar.2021.788524)
Supplement: Supplementary file 1 [file DataSheet1.docx]

**APPENDIX**

*Supplementary Table 1. Clinical improvement after treatment, postsurgical subgroup.*

| n total = 27 | ***Pain Improvement in Response to Treatment*** | | | |
| --- | --- | --- | --- | --- |
|  | **None (n; % of subgroup)** | **Mild (n; % of subgroup)** | **Moderate**  **(n; % of subgroup)** | **Marked**  **(n; % of subgroup)** |
| Improved with treatment (Y/N) | 16 (59.3%) | 4 (14.8%) | 3 (11.1%) | 4 (14.8%) |
|  | **None**  **(n; % of taken)** | **Mild**  **(n; % of taken)** | **Moderate**  **(n; % of taken)** | **Marked**  **(n; % of taken)** |
| **Oral Agents** | 16 (61.5%) | 3 (11.5%) | 3 (11.5%) | 4 (15.4%) |
| Pregabalin/Gabapentin | 10 (62.5%) | 1 (6.3%) | 1 (6.3%) | 4 (25%) |
| TCA (amitriptyline) | 3 (100%) | 0 (0%) | 0 (0%) | 0 (0%) |
| SNRI (duloxetine) | 4 (80%) | 0 (0%) | 0 (0%) | 1 (20%) |
| Anticonvulsant (topiramate) | 2 (66.7%) | 0 (0%) | 1 (33.3%) | 0 (0%) |
| Acetaminophen | 2 (40%) | 2 (40%) | 0 (0%) | 1 (20%) |
| Any NSAID* | 4 (66.7%) | 1 (16.7%) | 0 (0%) | 1 (16.7%) |
| Any muscle relaxant† | 1 (100%) | 0 (0%) | 0 (0%) | 0 (0%) |
| Any opioid agonist/antagonist** | 3 (100%) | 0 (0%) | 0 (0%) | 0 (0%) |
| **Topical Agents** | 9 (56.3%) | 3 (18.8%) | 1 (6.3%) | 3 (18.8%) |
| AST | 3 (37.5%) | 1 (12.5%) | 1 (12.5%) | 3 (37.5%) |
| Topical steroid | 1 (33.3%) | 1 (33.3%) | 1 (33.3%) | 0 (0%) |
| Topical cyclosporine, lifitegrast | 2 (28.6%) | 2 (28.6%) | 1 (14.3%) | 2 (28.6%) |
| Topical tacrolimus | 0 (0%) | 0 (0%) | 0 (0%) | 1 (100%) |
| **Adjuvant Agents** | 5 (45.5%) | 2 (18.2%) | 3 (27.3%) | 1 (9.1%) |
| TNS | 3 (60%) | 1 (20%) | 1 (20%) | 0 (0%) |
| Peri-ocular nerve block | 5 (55.6%) | 1 (11.1%) | 2 (22.2%) | 1 (11.1%) |
| Ganglion block | 1 (100%) | 0 (0%) | 0 (0%) | 0 (0%) |
| Botulinum injection | 0 (0%) | 0 (0%) | 0 (0%) | 0 (0%) |

*Ibuprofen, Diclofenac, Meloxicam, Celecoxib

†Baclofen, Cyclobenzaprine

**Tramadol, Naltrexone, Oxycodone

*TCA=tricyclic antidepressant; SNRI=serotonin-norepinephrine reuptake inhibitor; NSAID=Non-Steroidal Anti-Inflammatory Drug; AST=autologous serum tears; TNS=Trigeminal nerve stimulation*

*Supplementary Table 2. Clinical improvement after treatment, post-traumatic subgroup.*

| n total = 17 | ***Pain Improvement in Response to Treatment*** | | | |
| --- | --- | --- | --- | --- |
|  | **None (n; % of subgroup)** | **Mild (n; % of subgroup)** | **Moderate**  **(n; % of subgroup)** | **Marked**  **(n; % of subgroup)** |
| Improved with treatment (Y/N) | 4 (23.5%) | 8 (47.1%) | 3 (17.7%) | 2 (11.8%) |
|  | **None (n; % of taken)** | **Mild**  **(n; % of taken)** | **Moderate**  **(n; % of taken)** | **Marked**  **(n; % of taken)** |
| **Oral Agents** | 3 (18.8%) | 8 (50%) | 3 (18.8%) | 2 (12.5%) |
| Pregabalin/Gabapentin | 3 (27.3%) | 4 (36.4%) | 3 (27.3%) | 1 (9.1%) |
| TCA (amitriptyline) | 0 (0%) | 2 (66.7%) | 1 (33.3%) | 0 (0%) |
| SNRI (duloxetine) | 0 (0%) | 1 (33.3%) | 2 (66.7%) | 0 (0%) |
| Anticonvulsant (topiramate) | 0 (0%) | 1 (100%) | 0 (0%) | 0 (0%) |
| Acetaminophen | 0 (0%) | 2 (66.7%) | 1 (33.3%) | 0 (0%) |
| Any NSAID* | 2 (28.6%) | 3 (42.9%) | 2 (28.6%) | 0 (0%) |
| Any muscle relaxant† | 1 (50%) | 0 (0%) | 1 (50%) | 0 (0%) |
| Any opioid agonist/antagonist** | 0 (0%) | 2 (66.7%) | 1 (33.3%) | 0 (0%) |
| **Topical Agents** | 4 (33.3%) | 6 (50%) | 1 (8.3%) | 1 (8.3%) |
| AST | 1 (20%) | 3 (60%) | 1 (20%) | 0 (0%) |
| Topical steroid | 3 (100%) | 0 (0%) | 0 (0%) | 0 (0%) |
| Topical cyclosporine, lifitegrast | 0 (0%) | 1 (50%) | 0 (0%) | 1 (50%) |
| Topical tacrolimus | 0 (0%) | 3 (100%) | 0 (0%) | 0 (0%) |
| **Adjuvant Agents** | 2 (28.6%) | 4 (57.1%) | 1 (14.3%) | 0 (0%) |
| TNS | 1 (100%) | 0 (0%) | 0 (0%) | 0 (0%) |
| Periocular nerve block | 0 (0%) | 4 (80%) | 1 (20%) | 0 (0%) |
| Ganglion block | 0 (0%) | 0 (0%) | 1 (100%) | 0 (0%) |
| Botulinum injection | 0 (0%) | 0 (0%) | 0 (0%) | 0 (0%) |

*Ibuprofen, Diclofenac, Meloxicam, Celecoxib

†Baclofen, Cyclobenzaprine

**Tramadol, Naltrexone, Oxycodone

*TCA=tricyclic antidepressant; SNRI=serotonin-norepinephrine reuptake inhibitor; NSAID=Non-Steroidal Anti-Inflammatory Drug; AST=autologous serum tears; TNS=Trigeminal nerve stimulation*

*Supplementary Table 3. Clinical improvement after treatment, migraine-like subgroup.*

| n total = 41 | ***Pain Improvement in Response to Treatment*** | | | |
| --- | --- | --- | --- | --- |
|  | **None (n; % of subgroup)** | **Mild (n; % of subgroup)** | **Moderate**  **(n; % of subgroup)** | **Marked**  **(n; % of subgroup)** |
| Improved with treatment (Y/N) | 11 (26.8%) | 15 (36.6%) | 6 (14.6%) | 9 (22%) |
|  | **None (n; % of taken)** | **Mild**  **(n; % of taken)** | **Moderate**  **(n; % of taken)** | **Marked**  **(n; % of taken)** |
| **Oral Agents** | 10 (27%) | 14 (37.8%) | 5 (13.5%) | 8 (21.6%) |
| Pregabalin/Gabapentin | 6 (35.3%) | 6 (35.3%) | 1 (5.9%) | 4 (23.5%) |
| TCA (amitriptyline) | 0 (0%) | 0 (0%) | 0 (0%) | 0 (0%) |
| SNRI (duloxetine) | 3 (42.9%) | 2 (28.6%) | 1 (14.3%) | 0 (0%) |
| Anticonvulsant (topiramate) | 1 (33.35) | 0 (0%) | 0 (0%) | 2 (66.7%) |
| Acetaminophen | 5 (71.4%) | 1 (14.3%) | 1 (14.3%) | 0 (0%) |
| Any NSAID* | 3 (25%) | 4 (33.3%) | 3 (25%) | 2 (16.7%) |
| Any muscle relaxant† | 9 (34.6%) | 7 (26.9%) | 3 (11.5%) | 7 (26.9%) |
| Any opioid agonist/antagonist** | 2 (40%) | 2 (40%) | 0 (0%) | 1 (20%) |
| **Topical Agents** | 3 (21.4%) | 4 (28.6%) | 4 (28.6%) | 3 (21.4%) |
| AST | 0 (0%) | 2 (66.7%) | 1 (33.3%) | 0 (0%) |
| Topical steroid | 2 (33.3%) | 2 (33.3%) | 1 (16.7%) | 1 (16.7%) |
| Topical cyclosporine, lifitegrast | 2 (33.3%) | 2 (33.3%) | 1 (16.7%) | 1 (16.7%) |
| Topical tacrolimus | 0 | 1 (33.3%) | 1 (33.3%) | 1 (33.3%) |
| **Adjuvant Agents** | 4 (26.7%) | 6 (40%) | 2 (13.3%) | 3 (20%) |
| TNS | 3 (33.3%) | 3 (33.3%) | 1 (11.1%) | 2 (22.2%) |
| Peri-ocular nerve block | 1 (16.7%) | 2 (33.3%) | 2 (33.3%) | 1 (16.7%) |
| Ganglion block | 0 (0%) | 1 (100%) | 0 (0%) | 0 (0%) |
| Botulinum injection | 2 (20%) | 4 (40%) | 1 (10%) | 3 (30%) |

*Ibuprofen, Diclofenac, Meloxicam, Celecoxib

†Baclofen, Cyclobenzaprine

**Tramadol, Naltrexone, Oxycodone

*TCA=tricyclic antidepressant; SNRI=serotonin-norepinephrine reuptake inhibitor; NSAID=Non-Steroidal Anti-Inflammatory Drug; AST=autologous serum tears; TNS=Trigeminal nerve stimulation*

*Supplementary Table 4. Clinical improvement after treatment, unilateral subgroup.*

| n total = 16 | ***Pain Improvement in Response to Treatment*** | | | |
| --- | --- | --- | --- | --- |
|  | **None (n; % of subgroup)** | **Mild (n; % of subgroup)** | **Moderate**  **(n; % of subgroup)** | **Marked**  **(n; % of subgroup)** |
| Improved with treatment (Y/N) | 5 (31.3%) | 8 (50%) | 3 (18.8%) | 0 (0%) |
|  | **None (n; % of taken)** | **Mild**  **(n; % of taken)** | **Moderate**  **(n; % of taken)** | **Marked**  **(n; % of taken)** |
| **Oral Agents** | 3 (27.3%) | 6 (54.5%) | 2 (18.2%) | 0 (0%) |
| Pregabalin/Gabapentin | 1 (16.7%) | 4 (66.6%) | 1 (16.7%) | 0 (0%) |
| TCA (amitriptyline) | 1 (33.3%) | 1 (33.3%) | 1 (33.3%) | 0 (0%) |
| SNRI (duloxetine) | 2 (100%) | 0 (0%) | 0 (0%) | 0 (0%) |
| Anticonvulsant (topiramate) | 1 (50%) | 1 (50%) | 0 (0%) | 0 (0%) |
| Acetaminophen | 0 (0%) | 1 (50%) | 1 (50%) | 0 (0%) |
| Any NSAID* | 1 (14.3%) | 6 (85.7%) | 0 (0%) | 0 (0%) |
| Any muscle relaxant† | 1 (50%) | 1 (50%) | 0 (0%) | 0 (0%) |
| Any opioid agonist/antagonist** | 0 (0%) | 2 (50%) | 2 (50%) | 0 (0%) |
| **Topical Agents** | 3 (30%) | 4 (40%) | 3 (30%) | 0 (0%) |
| AST | 1 (20%) | 3 (60%) | 1 (20%) | 0 (0%) |
| Topical steroid | 3 (37.5%) | 4 (50%) | 1 (12.5%) | 0 (0%) |
| Topical cyclosporine, lifitegrast | 2 (66.7%) | 1 (33.3%) | 0 (0%) | 0 (0%) |
| Topical tacrolimus | 1 (50%) | 0 (0%) | 1 (50%) | 0 (0%) |
| **Adjuvant Agents** | 0 (0%) | 4 (66.7%) | 2 (33.3%) | 0 (0%) |
| TNS | 0 (0%) | 1 (100%) | 0 (0%) | 0 (0%) |
| Peri-ocular nerve block | 0 (0%) | 4 (80%) | 1 (20%) | 0 (0%) |
| Ganglion block | 0 (0%) | 2 (66.7%) | 1 (33.3%) | 0 (0%) |
| Botulinum injection | 0 (0%) | 1 (100%) | 0 (0%) | 0 (0%) |

*Ibuprofen, Diclofenac, Meloxicam, Celecoxib

†Baclofen, Cyclobenzaprine

**Tramadol, Naltrexone, Oxycodone

*TCA=tricyclic antidepressant; SNRI=serotonin-norepinephrine reuptake inhibitor; NSAID=Non-Steroidal Anti-Inflammatory Drug; AST=autologous serum tears; TNS=Trigeminal nerve stimulation*
